# Robustness of heart dose against cardiac cycle in breast cancer radiotherapy with deep inspiration breath-hold

**DOI:** 10.1007/s12194-026-01016-2

**Published:** 2026-02-08

**Authors:** Yuya Yanagi, Hajime Monzen, Ken Aoki, Naoki Harada, Kohei Ohashi, Marika Hayashi, Hiroyuki Kosaka, Harumi Iguchi

**Affiliations:** 1 Department of Radiology, Shiga University of Medical Science Hospital, Setatsukinowa, Otsu, Shiga 5202192 Japan; 2 Department of Medical Physics, Graduate School of Medical Sciences, Kindai University Osaka Medical Campus, 1-14-1 Miharadai, Minami-ku, Sakai, Osaka 5900197 Japan

**Keywords:** Deep inspiration breath-hold (DIBH), Left-sided breast cancer, Cardiac cycle, Heart dose management, Electrocardiogram-gated imaging

## Abstract

This study evaluated dose differences to the heart, left anterior descending coronary artery (LADCA), and left main coronary artery (LMCA) between diastolic and systolic heart phases in radiation therapy for left-sided breast cancer using deep inspiration breath-hold (DIBH). Diastolic and systolic doses to the heart, LADCA, and LMCA were analyzed using electrocardiogram-gated cardiac computed tomography images from 15 women. Radiation therapy plans were created for a total dose of 50 Gy in 25 fractions. Parameters assessed included volume, D_mean_, D_2%_, V_5Gy_, V_10Gy_, V_20Gy_, and V_25Gy_ for the heart; D_mean_, D_2%_, V_5Gy_, V_10Gy_, and V_20Gy_ for the LADCA; and D_mean_ and D_2%_ for the LMCA. The D_mean_ of the heart was 5.10 ± 3.04 Gy and 5.03 ± 3.05 Gy for diastole and systole, respectively (mean ± 1 standard deviation), and D_2%_ was 37.44 ± 16.03 Gy and 36.15 ± 16.76 Gy. Statistically significant differences were found in the D_mean_. LADCA doses showed no significant differences, possibly due to anatomical variations. The D_mean_ of the LMCA was 1.88 ± 0.23 Gy and 2.02 ± 0.28 Gy for diastole and systole, and D_2%_ was 2.05 ± 0.28 Gy and 2.21 ± 0.30 Gy, with both parameters being statistically significantly higher during systole. Although small, cardiac-phase-dependent dose variations under DIBH were statistically significant, confirming that current non-ECG-gated DIBH remains adequate for cardiac dose management.

## Introduction

The global incidence of breast cancer in young women has been increasing over the past decade [1]. However, improved survival rates among adolescents and young adults are associated with the risk of long-term late effects, including infertility, sexual dysfunction, cardiovascular disease, and secondary cancers, making long-term follow-up essential [2–4]. Additionally, cardiovascular disease is currently the leading cause of death among cancer survivors [5]. Increased cardiac mortality and morbidity from breast cancer treatment may be caused by both radiation therapy and drug therapy.

Breast cancer is now subtyped according to the nature of the cancer cells, with treatment methods tailored to each type. Anti-HER2 therapy, especially for HER2-positive types, is a cornerstone of treatment, although it is associated with high cardiotoxicity [6]. Along with drug therapy, radiation therapy following breast-conserving surgery is proven to reduce the local recurrence rate and should not be omitted [7–10]. The increased risk of cardiac mortality and morbidity due to radiation therapy has been reported to be dose-dependent [11, 12]. Sardaro et al. analyzed the mean heart dose received by women and reported that the risk of heart disease increased with the dose, estimating a 4% increase in heart disease risk for each gray rise in the mean heart dose [11]. Darby et al. stated that rates of major coronary events increased linearly with the mean dose to the heart, by 7.4% per Gy, with there being no apparent threshold and this increase being independent of the presence of preexisting cardiac risk factors [12]. While ischemic heart disease has been reported to decrease over time with advanced irradiation techniques [13, 14], it was shown that there is potentially an increased risk of heart failure and/or cardiomyopathy with left breast radiation therapy, highlighting the need for further clarification [15–18]. For these reasons, in radiation therapy after breast-conserving surgery, it is essential to reduce the absorbed dose to the heart and manage the dose accurately [18] through patient positioning [19] and intensity modulated radiation therapy (IMRT) [20]. The deep inspiration breath-hold (DIBH) technique was devised as an approach to reduce the dose to the heart without compromising target coverage, and its utility has been reported [21–24]. Additionally, recent reports suggest that motion management using a surface-guided radiation therapy system enhances DIBH stability [25, 26].

Although previous studies have presented appropriate margin to the heart and coronary arteries [27–30], detailed studies quantifying the difference in absorbed dose at different stages of the cardiac cycle are lacking. This study investigated these variations in the organs at risk (OARs), specifically the heart, left anterior descending coronary artery (LADCA), and left main coronary artery (LMCA), by utilizing diastolic and systolic images acquired via electrocardiographic (ECG)-gated cardiac computed tomography (CT). While ECG-synchronized beam delivery is not recommended for routine use, quantifying the exact dosimetric impact of cardiac motion is essential for validating the robustness of current treatment approaches. Therefore, we aimed to determine whether these cardiac-phase-dependent dose differences are clinically significant enough to justify the consideration of ECG-gated techniques.

## Materials and methods

### Patients and CT simulation

This study was approved by our institutional ethics review board. The study included 15 women who underwent coronary CT examinations with contrast enhancement at our hospital between February 2022 and December 2023. Retrospective analysis was performed on datasets where more than one cardiac cycle was imaged. The mean age was 63.5 years (range: 30–84) and the median age was 70 years. All patients were considered for radiotherapy planning with the DIBH technique because deep inspiration breath-hold imaging was performed. Patients were scanned in the supine position with arms raised above the head. The CT scanner used was an Aquilion ONE GENESIS edition (Canon Medical Systems, Tochigi, Japan), with a gantry rotation speed of 0.275 s and a slice thickness of 0.5 mm. Data were acquired using retrospective ECG gating. The systolic and diastolic phases were defined as 30–40% and 65–75% of the R–R interval, respectively. For both phases, image datasets were reconstructed using a half-scan method to improve temporal resolution during cardiac motion.

### Volume delineation

**Fig. 1:**
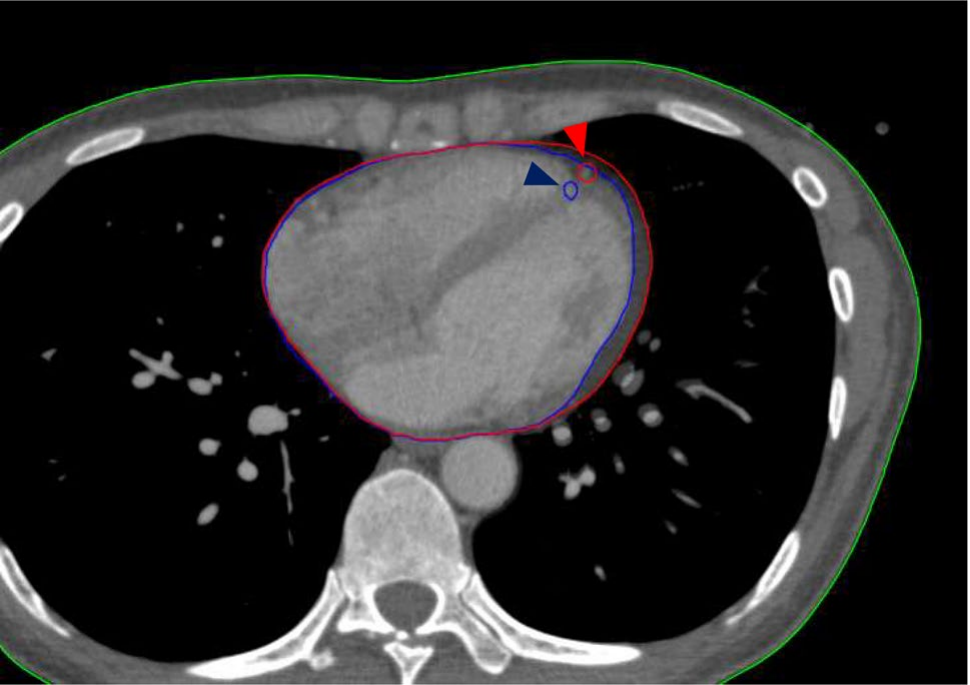
Representative computed tomography (CT) images illustrating the morphological differences between the diastolic and systolic phases in a single patient. The red contour indicates the diastolic phase, and the blue contour indicates the systolic phase. Triangles mark the location of the left anterior descending coronary artery (LADCA)

All OARs were contoured using an Eclipse treatment planning system (Varian Medical Systems, Palo Alto, CA, USA) according to the University of Michigan Cardiac Atlas proposed by Feng et al. [31]. Contour delineation of the OARs was performed manually by an experienced radiation oncologist. The heart, LADCA, and LMCA were designated as OARs. The heart contour included the entire pericardium from the lower left pulmonary artery to the apex, and the LADCA was contoured with a 5 mm diameter. The contour of the main trunk of the left coronary artery, originating from the left side of the ascending aorta, was delineated as the LMCA [31]. To visually support the observed morphological changes, a representative case illustrating the distinct contours in both the diastolic and systolic phases is presented in Fig. 1.

### Planning and dosimetric comparison

All plans were created using the Eclipse treatment planning system. Dose calculations were performed using the Acuros XB algorithm (version 13.5) with a grid size of 0.1 cm, reporting dose-to-medium. For the irradiation of the left breast, a standard plan of 50 Gy in 25 fractions was implemented using a tangential irradiation field that included a line connecting the axillary midline and the midline within the scanning range. To focus on the impact of cardiac motion, dose distribution was optimized using only physical wedges and a multi-leaf collimator; the field-in-field technique and bolus were not utilized. Radiation treatment plans were created and calculated using the same beam setup and monitor units for both diastolic and systolic phases within each patient. The mean heart dose (D_mean_; MHD), the dose absorbed by 2% of the organ (D_2%_), and the volumes of the organ receiving at least 5 Gy, 10 Gy, 20 Gy, and 25 Gy (V_5Gy_, V_10Gy_, V_20Gy_, V_25Gy_) for the heart; the D_mean_, D_2%_, V_5Gy_, V_10Gy_, and V_20Gy_ for the LADCA; and the D_mean_ and D_2%_ for the LMCA were compared for each patient between diastolic and systolic phases. Our comprehensive assessment included low-dose volume parameters based on the “no-threshold hypothesis” suggested by Darby et al., which implies that the risk of ischemic heart disease may accrue even from low-dose regions. This rationale ensures a thorough evaluation of dose uncertainty caused by cardiac motion [12].

### Statistics

Statistical evaluation was performed using SPSS version 25 (IBM Corp., Chicago, IL, USA). A two-tailed Wilcoxon signed-rank test was conducted to evaluate the dose parameters for the heart, LADCA, and LMCA, with *p*-values less than 0.05 being considered statistically significant.

## Results

**Fig. 2:**
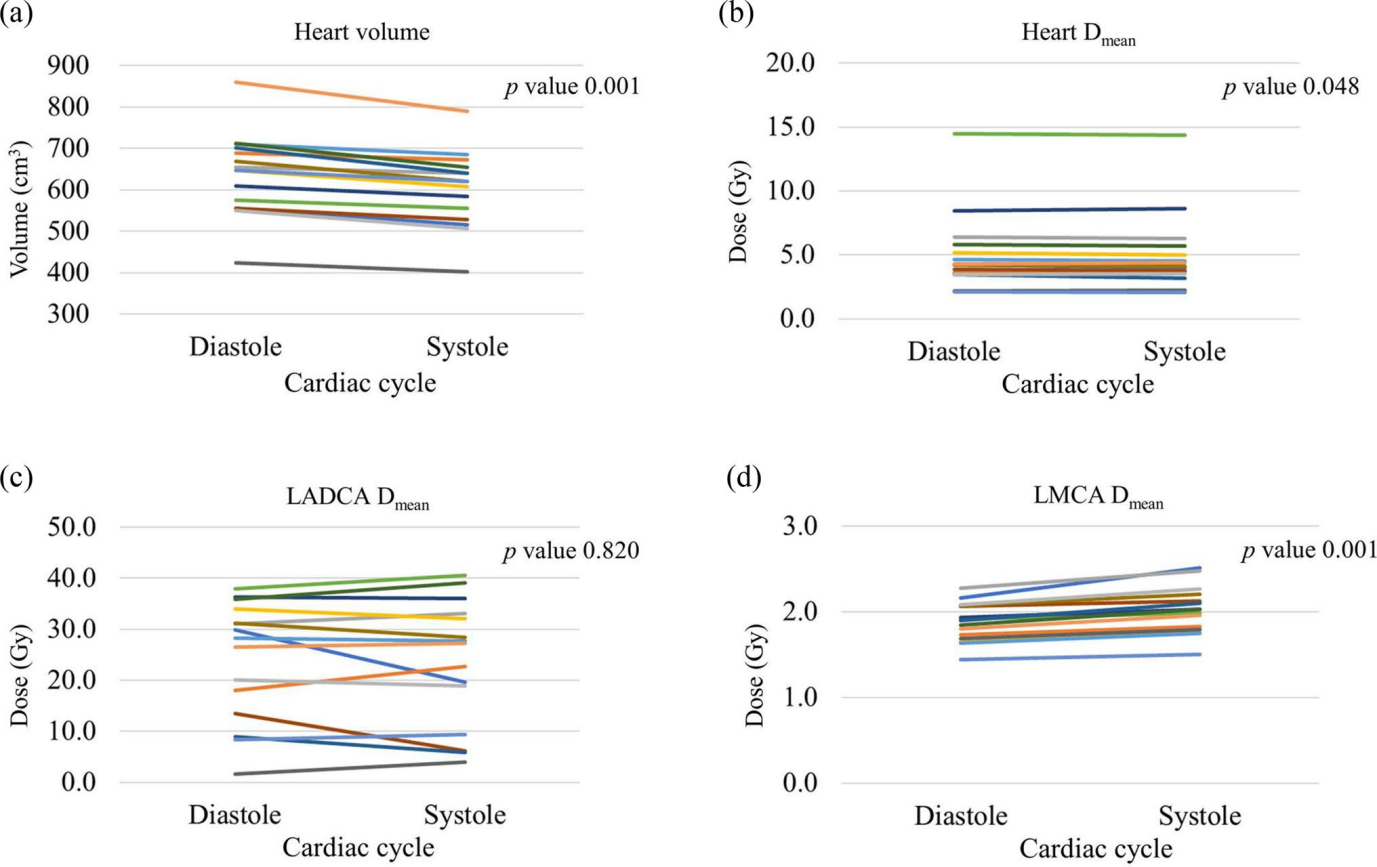
Cardiac phase-dependent variability in organ-at-risk (OAR) volume and D_mean_ between diastolic and systolic phases **a** heart volume variability, **b** D_mean_ comparison for the Heart, **c** D_mean_ comparison for the left anterior descending coronary artery (LADCA), and **d** D_mean_ comparison for the left main trunk coronary artery (LMCA)

The diastolic and systolic dose parameters are summarized in Table 1. For the heart, the mean heart volume was significantly smaller during systole (*p* < 0.01) (Fig. 2a). Dosimetrically, the D_mean_ was significantly lower during systole, whereas no statistically significant difference was observed for D_2%_. The distribution of D_mean_ differences for all heart cases is shown in Fig. 2b. Regarding volumetric parameters, no statistically significant difference was observed in the low-dose range of V_5Gy_; however, values for V_10Gy_, V_20Gy_, and V_25Gy_ were significantly lower during the systolic phase.

**Table 1:** Dose parameters for organs at risk in diastole and systole (mean ± 1 standard deviation)

Parameters	Diastole	Systole	*p*-value
Heart			
Volume (cm^3^)	637.4 ± 100.2	601.6 ± 91.6	0.001**
D_mean_ (Gy)	5.10 ± 3.04	5.03 ± 3.05	0.048*
D_2%_ (Gy)	37.44 ± 16.03	36.15 ± 16.76	0.219
V_5Gy_ (%)	11.47 ± 7.91	11.39 ± 7.91	0.447
V_10Gy_ (%)	7.58 ± 7.25	7.41 ± 7.27	0.038*
V_20Gy_ (%)	6.31 ± 6.75	6.13 ± 6.77	0.017*
V_25Gy_ (%)	5.85 ± 6.47	5.67 ± 6.50	0.018*
LADCA			
D_mean_ (Gy)	24.12 ± 11.58	23.40 ± 12.36	0.820
D_2%_ (Gy)	46.34 ± 11.66	43.57 ± 14.55	0.073
V_5Gy_ (%)	64.50 ± 14.60	63.78 ± 16.58	0.691
V_10Gy_ (%)	52.47 ± 21.97	49.86 ± 25.94	0.532
V_20Gy_ (%)	47.12 ± 24.05	44.65 ± 27.54	0.490
LMCA			
D_mean_ (Gy)	1.88 ± 0.23	2.02 ± 0.28	0.001**
D_2%_ (Gy)	2.05 ± 0.28	2.21 ± 0.30	< 0.001**

* < 0.05, ** < 0.01

For the LADCA, no significant difference was observed between diastole and systole for any of the parameters. This may be attributed to individual anatomical variations, although no specific contributing factors were identified in this study (Fig. 2c).

**Fig. 3:**
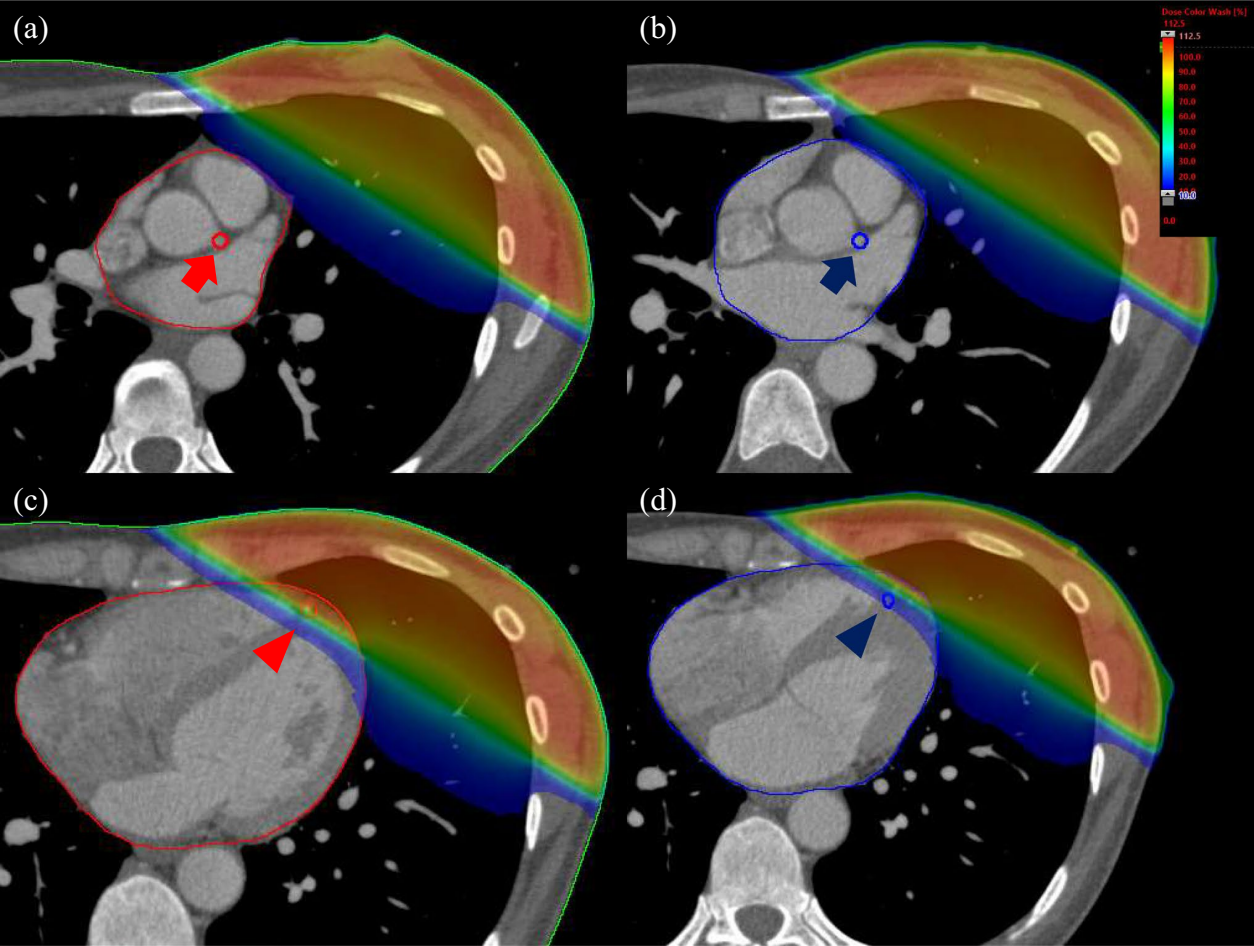
Dose distribution maps for the left main trunk coronary artery (LMCA) and the left anterior descending coronary artery (LADCA) in a single outlier patient. **a** LMCA origin level (diastolic phase), **b** LMCA origin level (systolic phase), **c** LADCA level (diastolic phase), and **d** LADCA level (systolic phase) (same level as (**c**)). The red contour indicates the diastolic phase, and the blue contour indicates the systolic phase. Arrow: LMCA origin; Triangle: LADCA

Conversely, for the LMCA, both D_mean_ and D_2%_ values were significantly higher during systole (*p* < 0.01). The distribution of D_mean_ differences for LMCA cases is presented in Fig. 2d. To visualize these findings, Fig. 3 illustrates representative dose distribution maps, highlighting the impact of cardiac motion on dose coverage for these structures in specific cases.

## Discussion

This study demonstrates that cardiac-phase-dependent dose variations under DIBH are clinically minimal, supporting the robustness of current non-ECG-gated practice. ECG-gated cardiac CT images of 15 women were used to create treatment plans simulating common radiation therapy after breast-conserving surgery (tangential radiation), and the doses received by the heart, LADCA, and LMCA during diastole and systole of the heart were evaluated in a comparative study. While radiation therapy after breast-conserving surgery reduces the local recurrence rate, the risk of cardiac disease due to cardiac exposure to ionizing radiation has been discussed. Accordingly, dose reduction to OARs has been achieved mainly through DIBH and optimized patient positioning [19–24]. However, the effect of the cardiac cycle under DIBH has not been estimated, and this is the first article to clarify the doses delivered to heart, LADCA, and LMCA during diastole and systole.

For the heart, the D_mean_ was 5.10 ± 3.04 Gy for diastole and 5.03 ± 3.05 Gy for systole, with significant differences between diastole and systole. In addition, the V_10Gy_, V_20Gy_, and V_25Gy_ of the heart were significantly lower in systole. This borderline significance further supports the minimal magnitude of the observed effect. The reason for this phenomenon is the effect of left ventricular variability during systole. Heart contraction is pronounced not only in the irradiated area, but also in the posterior area of the left ventricle, which is not irradiated. Therefore, the volume of the heart exposed to moderate doses in systole would have been markedly reduced compared with the D_mean_. Although significant differences were observed in these doses and volumes, the differences in their mean values were very small. Conversely, all parameters for LADCA were lower, but none showed significant differences. However, the inter-patient variability was substantial, with dose differences ranging widely from − 4.6 Gy to 10.2 Gy (standard deviation: ± 4.1 Gy) across the cohort. This is likely due to individual anatomical variations, as illustrated in Fig. 2c, with some patients exhibiting substantial dose reduction during systole (Fig. 3c, d). However, we did not find any factors specifically associated with this feature. For the LMCA, D_mean_ and D_2%_ were higher in systole in all patients. This may be due to a shift of the LMCA to the left anterior aspect due to cardiac contraction.

The relationship between cardiac exposure and risk of heart disease has been discussed in previous studies. Darby et al. reported a 7.4% increase in major coronary events for each 1 Gy increase in cardiac dose [12], whereas Holm et al. found no significant difference in cardiac ischemic events after radiation therapy for left and right breast cancer [32]. In addition, the dose absorbed by the heart may depend on ischemic events, and the risk of cardiac ischemic events has decreased over time [13, 14]. However, it has now been shown that there may be an increased risk of heart failure and/or cardiomyopathy with radiation therapy to the left breast, and dose reduction and dose management to the heart are considered increasingly important [15–18, 33]. Sardaro et al. estimated a 4% increase in risk of cardiac disease for every 1 Gy increase in mean cardiac dose [11], and Ritter et al. mentioned the risk of radiation-induced heart failure, pointing out the importance of accurate cardiac dose management [18].

Previous studies used cone beam CT to evaluate the planning organ at risk volume margin of the heart in DIBH and reported geometric uncertainties of 1–3 mm, 1–4 mm, and 2–7 mm in the left–right, anterior–posterior, and superior–inferior directions, respectively [28–30]. White et al. used cardiac-gated magnetic resonance imaging from 10 subjects to analyze LADCA motion blurring and simulate the dosimetric effect of overestimation of volume due to it, and found that it reduced the mean LADCA dose by an average of 23% ± 9% [27]. However, dose variations to OARs according to the phase of the cardiac cycle have not been previously reported. This study is the first to quantify cardiac-phase-dependent dose variations using ECG-gated cardiac CT data. These findings indicate that cardiac-phase-dependent dose variations were small under DIBH, suggesting that additional ECG gating provides limited clinical benefit in routine practice. We recognize that the absolute dose reduction of 0.07 Gy in the mean heart dose, while statistically detectable, is considered clinically negligible. Epidemiological evidence suggests that a meaningful reduction in long-term cardiac risk generally requires differences in the magnitude of several Gray. This indicates that the observed statistical differences do not provide a strong justification for the widespread clinical adoption of ECG-gated irradiation [11, 12].

Despite the minimal dose variations observed on average, our findings indicate the potential for significant localized dose reduction in specific high-risk patients. For instance, in a particular case, the mean dose to the LADCA drastically reduced from a maximum of 13.5 Gy in diastole to 6.2 Gy in systole, representing a substantial difference of 7.3 Gy (an approximately 54% reduction). Such a large variation is clinically critical, as it shifts the dose from a level that violates expert recommendations—specifically, the German Society for Radiation Oncology (DEGRO) consensus guidelines advise keeping the mean LADCA dose below 10 Gy, and ideally below 5 Gy, to minimize the risk of late cardiac toxicity [34]—to a level that is generally considered acceptable. This demonstrates that while the average benefit is small, ECG-gated irradiation retains a conceptual possibility for reducing critical OAR doses for specific patient subgroups, such as those at high risk of cardiac disease. Therefore, planners should be aware that OAR doses may be subject to these variations. Reis et al. also reported the technical feasibility of gating using real-time ECG signals [35, 36]. As an example of strict cardiac motion management in high-risk cardiac interventions, such techniques are already actively being researched in other fields, such as stereotactic arrhythmia radioablation (STAR) for ventricular tachycardia (VT) [36]. The feasibility of these techniques suggests that, while currently investigational, they could be adapted for breast cancer patients at high risk of cardiac disease. Although ECG-synchronized irradiation is not currently required for general clinical implementation, this study provides an important foundation for the future development of phase-aware radiotherapy.

This study has several limitations. First, the small sample size (15 patients) and the relative uniformity of the treatment plans used (simple tangential fields, 2 Gy per fraction) inherently limit the generalizability of our results. Future cohort studies, utilizing multi-institutional data with diverse equipment and advanced techniques such as field-in-field or volumetric modulated arc therapy (VMAT), are essential to fully validate our conclusions and to establish clear associations between cardiac dose variability and clinical factors (e.g., age, left ventricular ejection fraction (LVEF)). However, we anticipate that the impact of the cardiac cycle may be even smaller with advanced techniques like IMRT, which utilize steeper dose gradients, further emphasizing the sufficiency of non-ECG-gated DIBH in the modern radiotherapy era. A second major limitation is that the comparison was restricted to the extreme cardiac motion phases (diastole and systole) due to the technical inability to reconstruct images during typical 'free-beating' cardiac motion seen in clinical practice. This prevented a direct comparison with the dose delivered by regular non-gated CT. However, routine non-gated DIBH CT essentially captures an averaged state or a random instance within the cardiac cycle. Since our results demonstrated that the dose difference between the two most extreme phases (diastole and systole) was clinically negligible (0.07 Gy for MHD), the dose variation in routine non-gated CT is theoretically bounded within this minimal range. This finding strongly supports the current use of non-ECG-gated DIBH techniques as possessing sufficient robustness for routine cardiac dose management.

## Conclusions

Although small, cardiac-phase-dependent dose variations under DIBH were statistically significant, confirming that current non-ECG-gated DIBH remains adequate for cardiac dose management. Nevertheless, the substantial localized variations observed in selected cases suggest a conceptual possibility for achieving further dose reduction in high-risk patient subgroups through the future development of phase-aware delivery techniques.

## Data Availability

The data that support the findings of this study are available from the corresponding author upon reasonable request.
